# Management of Fibrosis: The Mesenchymal Stromal Cells Breakthrough

**DOI:** 10.1155/2014/340257

**Published:** 2014-07-14

**Authors:** Benoît Usunier, Marc Benderitter, Radia Tamarat, Alain Chapel

**Affiliations:** Radioprotection and Human Health Division, Institute of Radioprotection and Nuclear Safety, PRP-HOM/SRBE/LR2I, 92260 Fontenay-aux-Roses, France

## Abstract

Fibrosis is the endpoint of many chronic inflammatory diseases and is defined by an abnormal accumulation of extracellular matrix components. Despite its slow progression, it leads to organ malfunction. Fibrosis can affect almost any tissue. Due to its high frequency, in particular in the heart, lungs, liver, and kidneys, many studies have been conducted to find satisfactory treatments. Despite these efforts, current fibrosis management therapies either are insufficiently effective or induce severe adverse effects. In the light of these facts, innovative experimental therapies are being investigated. Among these, cell therapy is regarded as one of the best candidates. In particular, mesenchymal stromal cells (MSCs) have great potential in the treatment of inflammatory diseases. The value of their immunomodulatory effects and their ability to act on profibrotic factors such as oxidative stress, hypoxia, and the transforming growth factor-*β*1 pathway has already been highlighted in preclinical and clinical studies. Furthermore, their propensity to act depending on the microenvironment surrounding them enhances their curative properties. In this paper, we review a large range of studies addressing the use of MSCs in the treatment of fibrotic diseases. The results reported here suggest that MSCs have antifibrotic potential for several organs.

## 1. Introduction

Healthy tissues can be damaged under various conditions by acute or chronic stimuli such as mechanical or chemical injuries, infections, or autoimmune reactions. In most cases, the repair process consists of dead and damaged cells replacement, thus restoring the organ's unimpaired functionality. The first stage of this mechanism, known as the regenerative phase, corresponds to the replacement of damaged cells by cells of the same type, thus ensuring organ functionality. During the second phase, known as fibroplasia or fibrosis, connective tissue replaces degraded normal parenchymal tissue. Unchecked fibrosis leads to substantial remodeling of the ECM (extracellular matrix) with pathological features which results in the formation of permanent scar tissue. Fibrosis may ultimately lead to organ malfunction and death. It mainly originates from chronic inflammation, tissue ischemia, and imbalance in the ECM accumulation/degradation ratio [1].

**Figure 1:**
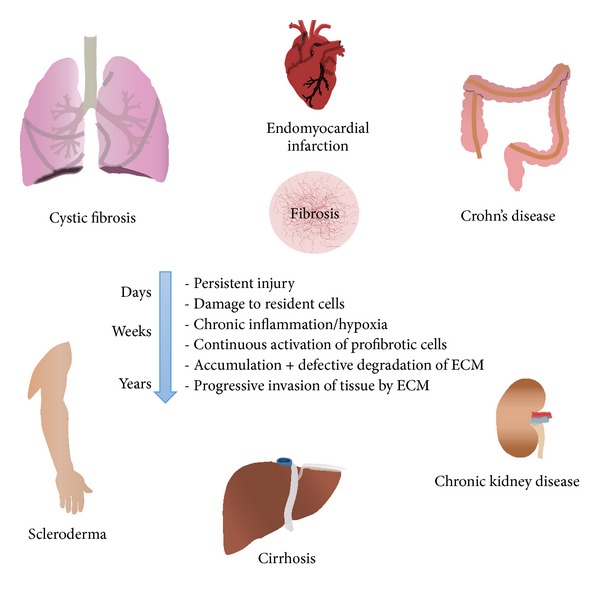
Fibrotic pathologies in various organs. Common features of fibrosis development and progression in various organs and related diseases (ECM: extracellular matrix).

Most organs are susceptible to fibrotic diseases, generally as a consequence or feature of a preexisting pathology (Figure 1). Obesity, aging, and environmental aggressions are the main causes of fibrogenesis. Fibroproliferative diseases are believed to be responsible for around 45% of deaths in developed countries [2]. Although considerable efforts are being devoted to the search for antifibrotic treatments, there are currently few effective therapies for fibrotic diseases that do not result in severe secondary effects. Anti-inflammatory drugs have been considered as the most promising candidates in clinical trials. A wide range of antioxidants have also been tested. Nevertheless, most drug therapy protocols have failed in achieving sufficient antifibrotic effect.

Thus, cell therapy has recently been put forward as a possibility. In particular, mesenchymal stromal cell (MSC) therapy seems to be a promising treatment. Indeed, preclinical and clinical trials have shown MSCs' ability to improve outcomes in various diseases such as the consequences of radiotherapy [3], autoimmune pathologies [4], neurodegenerative disorders [5], and other etiological agents. Preclinical and clinical studies have also put forward the ability of MSCs to adapt to their environment. Indeed, the regulation of MSCs' secretome is highly influenced by the surrounding tissue. Therefore, MSC therapy yields different results with different pathologies. Consequently, these effects have led several laboratories to investigate the antifibrotic potential of MSCs.

## 2. Common Cellular and Molecular Mechanisms of Fibrotic Diseases

### 2.1. Wound Healing: The Initiation of Fibrosis

Tissue injuries induce damages to resident cells which secrete inflammatory mediators which initiate an antifibrinolytic-coagulation cascade associated with vascular congestion. A temporary ECM is formed to serve as a scaffold for dead cells replacement. Subsequent platelet activation causes the release of various mediators including vasoactive factors (vasodilatation, increased vascular permeability, and edema by plasma exudation), cytokines, and chemokines that enable the recruitment of leukocytes. The formation of a fibrin clot serves as a matrix for cell migration and platelet adhesion. Fibrinolysis is later activated and leads to the dissolution of the fibrin clot replaced by a granulation tissue. Plasmin is released from the fibrin clot and activates the complement system, triggering the release of chemotactic and vasoactive anaphylatoxins [1, 6].

Next, recruited leukocytes home by adhesion to molecules such as selectins, integrins, and immunoglobulins. Phagocytosis of tissue debris, dead cells, and any exogenous organisms is carried out by macrophages and neutrophils. They also produce cytokines and chemokines to recruit endothelial cells necessary for neovascularization. The interaction of fibroblasts, fibrocytes, or other resident cells, such as hepatic stellate cells (HSCs), with the microenvironment induces their differentiation into myofibroblasts which synthesize ECM and growth factors including profibrotic TGF-*β*1 (transforming growth factor-*β*1). The secretion of autocrine hormones enables the maturation of myofibroblasts. *α*-SMA (*α*-smooth muscle actin) and vimentin expression by myofibroblasts are responsible for their contractile activity [7]. This contractibility is required for the closure of the wound. The formation of this so-called granulation tissue is characterized by the presence of many blood capillaries allowing the supply of nutrients, hormones, and respiratory gas [1, 2, 6].

Finally, the migration and maturation of epithelial and endothelial cells then allow the formation of scar tissue and neovascularization. The provisional ECM is degraded by matrix metalloproteinases (MMPs) once complete tissue replacement is achieved. The subtle equilibrium between MMPs and their inhibitors, tissue inhibitors of metalloproteinases (TIMPs), controls ECM accumulation and degradation throughout the repair process. Thus, it guarantees proper ECM remodeling by inducing a shift in matrix composition. Next, myofibroblasts disappear by apoptosis, triggered by the establishment of a negative activation loop indicating regeneration of the injured tissue [1, 2, 6].

### 2.2. Specific Fibrosis Mechanisms

Various fibroproliferative pathologies share common features. Fibrosis begins as a normal tissue regeneration process. Resident and recruited cells are activated to produce a provisional ECM facilitating repair. However, in the case of bacterial infection, ischemia, chronic inflammation, or other persistent stimuli, a constant loop of myofibroblast activation sets in, leading to excessive ECM accumulation. Activated myofibroblasts also produce chemokines to recruit cells from the immune system (macrophages, T- and B-cells, neutrophils, and eosinophils), thus perpetuating chronic inflammation. The pathologic matrix progressively invades the tissue, eventually ending in the presence of a permanent fibrotic scar. Histologically, fibrosis can be defined by two distinct stages. Development corresponds to the onset of matrix accumulation where only scattered fibrosis areas are seen in the tissue, whereas the endpoint is characterized by diffused spans of ECM possibly distributed through the entire tissue. The progressive replacement of dead cells by ECM suppresses organ function and induces stiffness. Ultimately, the best course of treatment for advanced fibrosis is often organ transplantation.

Fibrosis is a complex pathology driven by numerous biological factors such as chronic inflammation and hypoxia. Ionizing radiation, for example, induces endothelial cell death and oxidative stress, resulting in prolonged inflammation and potentially fibrosis. The constant recruitment of inflammatory cells generates an activation loop of myofibroblasts and maintains a steady pool of profibrotic cells.

One of the main molecular agents inducing fibrosis is TGF-*β*1, mainly synthesized by T-cells during the healing process [8]. TGF-*β*1 is secreted in a latent form associated with LAP (latency associated peptide). LAP is cleaved to allow the activation of TGF-*β*1 which is able to bind its receptors TGF-*β*R1 (transforming growth factor receptor-*β*1) and TGF-*β*R2. Therefore, there is a large pool of inactive TGF-*β*1 in the extracellular environment. Various agents can induce TGF-*β*1 activation: MMPs [9], reactive oxygen and nitrogen species (ROS and RNS) [10], cytokines [11], or other stimuli such as ionizing radiation [12]. The binding of TGF-*β*1 to its receptors activates the Smad (small mothers against decapentaplegic homolog) signaling pathway which induces the transcription of various genes, including genes encoding members of the extracellular matrix (collagens mostly) [13]. It also activates the differentiation of fibrocytes toward functional fibroblasts.

EMT (epithelial-to-mesenchymal transition) and EndMT (endothelial-to-mesenchymal transition) are also described as important sources of fibroblasts. Epithelial or endothelial cells assume a spindle shape, lose their cell markers, and express typical fibroblast markers such as FSP-1 (fibroblast specific protein-1), *α*-SMA, and vimentin [14, 15]. They also acquire the ability to produce collagen and fibronectin (extracellular matrix components) [16]. TGF-*β*1 has also been shown to decrease the expression and activity of MMPs and increase the expression of TIMPs [17]. Thus, TGF-*β*1 is considered to be one of the major factors in fibrosis development.

Other growth factors take part in prolonged fibrogenesis. CTGF (connective tissue growth factor) acts synergistically with TGF-*β*1 to stimulate the signal transduction pathway dependent on TGF-*β*1 [18]. CTGF can also stimulate the proliferation, migration, and adhesion of fibroblasts and the production of the extracellular matrix [19, 20].

**Figure 2:**
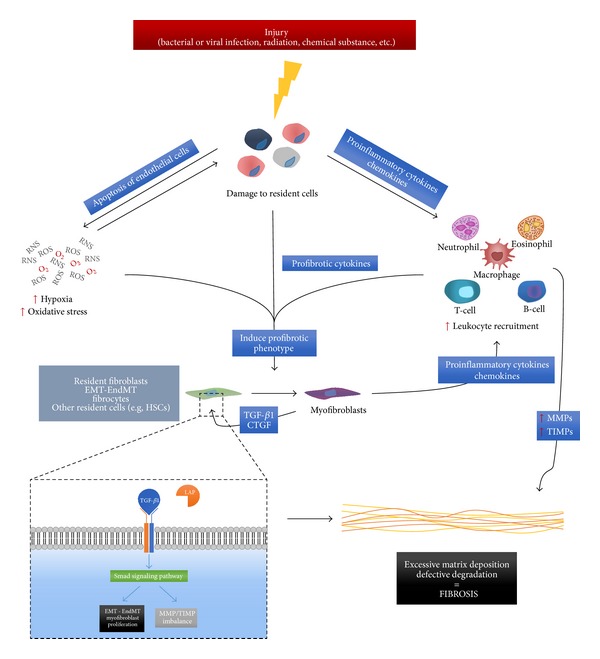
Fibrosis is a multicomponent pathology driven by multiple factors. Fibrotic diseases are driven by multiple factors such as inflammatory reaction, hypoxia, and oxidative stress leading to the activation of the TGF-*β*1 pathway (DC: dendritic cell, EMT: epithelial-to-mesenchymal transition, LAP: latency associated protein, MMP: matrix metalloproteinase, RNS: reactive nitrogen species, ROS: reactive oxygen species, Smad: small mothers against decapentaplegic homolog, TGF: transforming growth factor, and TIMP: tissue inhibitor of metalloproteinases).

Thus, fibrosis is a multicomponent pathology driven by multiple factors (Figure 2). One of the main issues in treating fibrosis lies in its self-maintenance. Hence, various therapies might be considered depending on the stage of fibrogenesis. Indeed, preventive or curative strategies should differ based on the ECM components and the mechanisms involved. Moreover, combined therapies should be used to simultaneously act on various profibrotic mechanisms and enhance treatment efficacy.

## 3. Fibrosis Models

Over the years, many models of fibrosis in animals have been developed. Mechanical or chemical procedures are used to mimic damage observed in patients.

*Heart.* Cardiac fibrosis is characteristic of many heart diseases. Doxorubicin (DOX) or isoproterenol (ISO) is widely used to induce myocardial infarction (MI). It is hypothesized that DOX-induced cardiac damage increases the concentration of reactive oxygen species, thus causing injury to mitochondria, leading to apoptosis and fibrosis [21]. ISO injection directly into the heart produces diffuse myocardial cell death and fibrosis, leading to progressive heart failure [22]. Finally, ligation of the interventricular artery results in ischemia and eventually leads to fibrosis [23].

*Kidney.* Interstitial fibrosis and glomerulosclerosis are common features of kidney pathologies such as chronic kidney disease (CKD), chronic allograft nephropathy (CAN), or ureteral obstruction. In the reversible unilateral ureteral obstruction (UUO), fibrosis is induced by oxidative stress [24]. Atherosclerotic renal artery stenosis (ARAS) is found among 50% of atherosclerotic patients with other atherosclerotic diseases [25]. In preclinical studies, ARAS is modeled by placing an irritant coil in one of the main renal arteries to induce chronic inflammation [26]. Removal of one or both kidneys and kidney allograft can be performed to create a CAN model [27]. “Nephrectomy + ischemia-reperfusion + cyclosporine” (NIRC) is a recent model mimicking CKD. Oxidative stress caused by ischemia, exacerbated by the immunosuppressive effect of cyclosporine, induces interstitial fibrosis following ischemia-reperfusion [28]. Lastly, in the remnant kidney model (RKM), also called 5/6 nephrectomy (5/6 NX), interstitial fibrosis is induced by removing one kidney and two-thirds of the second. It is hypothesized that subsequent oxidative stress and inflammatory reaction generate fibrosis [29].

*Liver.* Fibrosis in the liver, or cirrhosis, is the common endpoint of chronic liver diseases. It originates from not only numerous pathologies such as alcoholic liver disease and viral or autoimmune hepatitis but also hepatotoxic drugs and toxins. Carbon tetrachloride (CCl_4_) induces irreversible pathologies such as fatty liver, fibrosis, cirrhosis, and cancer and is mainly used in liver damage models [30].

*Lungs.* Pulmonary fibrosis is an increasingly frequent pathology due to the growing number of smokers and the pollution resulting from current lifestyles. The onset of fibrosis in the bleomycin, mainly originating from DNA single and double strand breaks, is a major side effect of this drug which is now widely used in the development of animal models of pulmonary fibrosis [31]. Exposure to silica also induces fibrotic responses. The resulting persistent toxic effect causes chronic inflammation resulting in fibrogenesis [32].

*Peritoneum.* Peritoneal fibrosis can be initiated by toxins, infectious peritonitis, or incompatible dialysate products. Chlorhexidine gluconate (CG) was one of the first compounds believed to cause encapsulating peritoneal sclerosis (EPS) during dialysis. Peritoneal exposure to CG leads to an inflammatory reaction causing fibrosis in animal models [33].

*Skin.* Skin fibrosis is part of a wide range of human disorders including keloids, hypertrophic scars, and scleroderma. Subcutaneous injections of bleomycin produce lesions mimicking scleroderma [34]. Radiation exposure can lead to fibrosis in a number of different organs. Cutaneous radiation-induced fibrosis is caused by a strong inflammatory reaction, apoptosis, and oxidative stress and is a commonly used animal model [35]. Another* in vivo* cutaneous fibrosis model has been developed in mice by producing full-thickness wounds which consequently lead to chronic inflammation [36, 37].

*Pancreas.* The incidence of chronic pancreatitis is approximately 30 per 100,000 and is increasing over time [38]. Since existing treatments are limited, continuous efforts are being devoted to preclinical studies in animal models. Intravenous administration of dibutyltin dichloride (DBTC) induces damage to the bile duct epithelium. Subsequent inflammation causes fibrosis in the pancreas [39].

*Colon-Rectum.* 5 to 10% of patients receiving pelvic radiotherapy develop chronic radiation proctopathy due to the high radiosensibility of organs in the radiation field (colon, rectum, and bladder) [3]. Radiation proctopathy is modeled in animals by delivering a high radiation dose to the rectum [40]. Radiation-induced damage to the tissue as well as oxidative stress induces fibrosis in this model.

Common features are characteristic of these animal models of fibrosis. Chemical compounds, physical agents, or surgery procedures are used to induce the initial injury. This protocol is often repeated periodically or maintained over a prolonged time. Subsequent damage to the tissue induce chronic inflammation, oxidative stress, and/or hypoxia necessary to activate resident and recruited cells toward a profibrotic phenotype. In most cases, fibrotic features appear weeks to months after the initial stimulus.

## 4. Antifibrotic Effects of Mesenchymal Stromal Cells Therapy

MSCs are widely described for their immunoregulatory properties. Nevertheless MSCs' antifibrotic functions are poorly described. Syntheses of* in vivo* study outcomes are described in Table 1 (heart), Table 2 (liver), Table 3 (kidneys), Table 4 (lungs), Table 5 (peritoneum), Table 6 (pancreas), Table 7 (skin), and Table 8 (rectum). The synthesis of* in vitro* study outcomes is shown in Table 9.

### 4.1. Immunological Aspects

**Figure 3:**
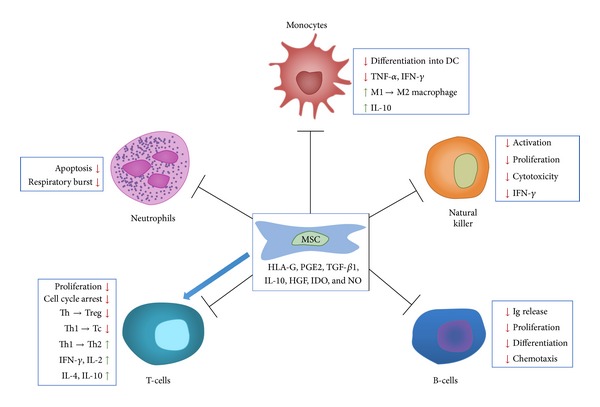
MSCs exert various effects on immune cells. A summary of MSC-mediated effects on the immune response. Various factors secreted by MSC exert an inhibitory effect on cells of the immune system which are involved in the fibrotic process (HGF: hepatocyte growth factor, HLA: human leukocyte antigen, IDO: indoleamine 2,3-dioxygenase, IFN-*γ*: interferon-*γ*, Ig: immunoglobulin, IL: interleukin, MSC: mesenchymal stromal cell, NO: nitric oxide, PGE2: prostaglandin E2, Tc: cytotoxic T-cell, TGF-*β*: transforming growth factor-*β*, TNF-*α*: tumor necrosis factor-*α*, Th: helper T-cell, and Treg: regulatory T-cell).

Pathogenic fibrosis results from chronic inflammatory reactions. Recent advances in the immunobiology of MSCs have led to increased interest in their use as a new therapeutic modality to address chronic inflammation associated with fibrosis (Figure 3) [78, 79]. The immunosuppressive effect of MSCs has been extensively studied and documented, particularly because of its value in organ transplantation. MSCs operate on the T and B lymphocytes by blocking them in the G0/G1 phase of the cell cycle, inhibiting the production of immunoglobulins (IgA, IgG, and IgM) and the differentiation of B lymphocytes. MSCs induce a change in polarity in T lymphocytes from a proinflammatory Th1 state to an anti-inflammatory Th2 condition [80, 81]. They act in the differentiation and maturation of dendritic cells and make them tolerogenic [82]. Furthermore, MSCs inhibit the cytotoxic activity of natural killer cells on HLA-1 (human leukocyte antigen-1) negative cells and reduce the production of cytokines: TNF-*α* (tumor necrosis factor-*α*), IFN-*γ* (interferon-*γ*), and IL-10 (interleukin-10) [83]. Therefore, MSCs are of value for the treatment of diseases with an inflammatory component.

Numerous studies have highlighted the benefits of immunomodulation by MSCs in the treatment of fibrosis. MSC-induced decreased TLR (toll-like receptor) expression suggests their ability to limit chronic inflammation [40]. After the transplantation of MSCs, a decreased infiltration of monocytes/macrophages, neutrophils, and lymphocytes in the tissue was observed in various models [40, 69, 70, 73, 74]. This correlates with the decreased expression of MCP-1 (monocyte chemoattractant protein-1) in some cases [74]. Additionally, underexpression of VCAM-1 (vascular cell adhesion molecule-1) and ICAM-1 (intercellular adhesion molecule-1), involved in leukocyte-endothelial cell interactions, suggests reduced inflammatory cell infiltration [74]. In a model of radiation-induced skin fibrosis, MSCs induced macrophage transition toward a regulatory phenotype, thus limiting chronic inflammation causing fibrosis [76]. Decreased iNOS (nitric oxide synthase) expression after MSC transplantation suggests a reduction of M1 macrophage activity [40]. An increased proportion of anti-inflammatory M2 macrophages were reported after MSC transplantation in a heart fibrosis model [45] and a radiation-induced proctitis model [40]. Microvesicles purified from MSC-conditioned medium, while significantly decreasing the amount of inflammatory cells, produced lower effects compared to MSC transplantation in the lung [69].

MSCs inhibit the expression of IFN-*γ*, which exerts a proinflammatory effect by inducing overexpression of IL-6 and TNF-*α* [67]. The decrease in mRNA expression and protein concentration of TNF-*α*, a profibrotic cytokine, was detected in the tissue after MSC transplantation [40, 58, 60, 65–67, 70, 74, 76, 77]. IL-1*α* [76], IL-1*β* [70, 76], and IL-6 [40, 63, 65, 70, 74] are underexpressed in several fibrosis models following MSC injection. Increased expression of anti-inflammatory cytokines IL-4 and IL-10 after MSC transplantation was observed, suggesting the transition of T lymphocytes to a Th2 profile [65]. Similarly, MSCs induced increased IL-10 expression and concentration in a model of cutaneous and rectal radiation-induced fibrosis [40, 76].

Antiapoptotic effects of MSC therapy can also be discussed, as fewer apoptotic events correlate with reduced inflammation. In fibrotic tissues following MSC transplantation, a decrease in apoptotic events was observed [41, 42, 52, 65]. Accordingly, MSCs may protect resident cells, increasing functionality and recovery.

MSCs may induce regression in pathophysiological processes associated with fibrosis. These effects are in part mediated by a reduction in chronic inflammation. MSCs likely proceed by a change in immune cell function, an increase in anti-inflammatory cytokines, and a decrease in proinflammatory cytokines and cell apoptosis. These immune mechanisms contribute to a modification of the microenvironment, thus diminishing tissue fibrosis, increasing resident stem cell proliferation, and eventually leading to tissue regeneration.

### 4.2. The TGF-*β*1 Pathway

TGF-*β*1 has been described as one of the major players in fibrosis. Its binding to receptors induces the activation of a signaling cascade leading to the proliferation of phenotypically profibrotic cells such as myofibroblasts. In particular, it induces the EMT and EndMT in part responsible for the proliferation of cells synthesizing ECM. The TGF-*β*1 signaling pathway is one of the prime targets for antifibrotic therapies and its regulation has been abundantly studied in treatment trials with MSCs. Generally, MSC transplantation reduces the expression and concentration of TGF-*β*1 [40, 49, 59, 60, 65, 67, 70, 71, 73–76]. The same effect is induced by transplanting exosomes isolated from MSC-conditioned medium [47].* In vitro*, Ueno et al. showed the inhibition of TGF-*β*1 overexpression induced by glucose in a coculture model of MSCs and peritoneal mesothelial cells [73]. This effect was associated with the decrease in the phosphorylation of Smad-2, as also shown in an exosome transplantation model [47, 73]. Reduced expression of *α*-SMA [44, 48, 50, 51, 60, 65, 66, 73] and the lower number of *α*-SMA positive cells [52, 53, 59, 64, 74, 75] suggest a decrease in the proliferation of myofibroblasts and, to a lesser extent, of TGF-*β*1-mediated EMT.* In vitro*, a reduced concentration of *α*-SMA in a coculture of MSCs and HK2 (human kidney 2) cells pretreated with TGF-*β*1 suggests a direct effect by MSCs on phenotypic changes leading to the accumulation of profibrotic cells [64]. A decreased expression and concentration of CTGF in several models also participate in diminishing profibrotic cells proliferation [40, 63].

Interestingly, several studies have underlined the importance of HGF (hepatocyte growth factor) secreted by MSCs for their antifibrotic effects [44, 47, 73]. MSCs transfected with an HGF expression plasmid yielded better results than nontransfected MSCs in a pulmonary fibrosis model [72]. The use of recombinant HGF partially reproduced the effects of MSCs in a coculture model with albumin-treated proximal tubular epithelial cells (PTECs) [62]. The inhibition of TGF-*β*1 expression by HGF and its ability to ameliorate the degradation of collagen through the increase in MMP-1 concentration highlights the value of such therapy [84]. Moreover, the increased expression of p-Met, which induces the phosphorylation of c-Met, the HGF membrane receptor, is also part of the action mechanisms of MSCs [59].

Recently, Qi et al. highlighted the importance of TSG-6 (TNF-stimulated gene 6) in the antifibrotic effect of MSCs. In addition to suppressing the secretion of TNF-*α* by activated macrophages, this protein induces a change in the TGF-*β*1/TGF-*β*3 balance, from a profibrotic high ratio to an antifibrotic low ratio [77]. These results are confirmed in a coculture model in which recombinant TSG-6 partially reproduced the effects of MSCs [62].

### 4.3. Hypoxia/Oxidative Stress

Accumulation of ECM in the tissue, death of endothelial cells, and increased levels of reactive oxygen and nitrogen species (ROS and RNS, resp.) result in hypoxia and oxidative stress during fibrosis. These factors lead to increased apoptosis and activation of TGF-*β*1. The improved vascularization of tissue and a more effective neutralization of oxidizing radicals would therefore enhance the effectiveness of antifibrotic therapies.

MSCs' ability to relieve oxidative stress has already been shown in several works. First, they seem to increase the expression and concentration of enzymes responsible for scavenging free radicals, such as NQO1 (NADPH quinone oxidoreductase 1), Gr (glutathione reductase), GPx (glutathione peroxidase), and HO-1 (heme oxygenase 1) [85, 86]. Nrf2 (nuclear factor (erythroid-derived 2)-like 2) activation is protective against oxidative stress and induces SOD (superoxide dismutase) production which decreases ROS concentration in the liver. MSC treatment correlates with an increase in Nrf2 and SOD which might reduce ROS accumulation, thus decreasing oxidative stress [87]. In a coculture model, an increased survival of cerebellar neurons is correlated with the secretion of SOD3 by MSCs [88].

MSC-mediated angiogenesis has also been demonstrated. MSCs are able to secrete a large range of angiogenic factors such as VEGF (vascular endothelial growth factor), FGF-2 (fibroblast growth factor-2), and MCP-1 [89–91]. Some studies also suggest their ability to promote endothelial cell proliferation [92, 93]. The reduced expression of VEGF, associated with improved microcirculation in the tissue after MSC transplantation, was observed [53]. Mias et al. showed a stimulation of angiogenesis following treatment with MSCs [44]. The transplantation of MSC sheets into the scarred myocardium increased neovascularization in a myocardial infarction model [45]. The authors also reported evidences of MSCs differentiating to participate in the formation of new vascular structures.

Conversely, an increased expression of VEGF posttreatment, with the concomitant overexpression of HIF-1*α*, was shown in a renal fibrosis model, indicating elevated tissue hypoxia [61]. HIF-1*α* (hypoxia-inducible factor-1*α*) stimulates the expression of VEGF under hypoxic conditions. In the same way, in a radiation-induced proctitis model, the overexpression of VEGF was accompanied by a reduction in angiopoietin and PDGF expression [40]. It can be hypothesized that insufficient angiogenesis in these models induces these variations. This gene expression profile may reflect proangiogenic signals mediated by MSCs. The evaluation of tissue vascularization would give better insights into MSCs effect on angiogenesis in these models.

MSCs may therefore act in different ways on hypoxia and oxidative stress by increasing angiogenesis in the tissue and by improving the inactivation of ROS and RNS. This feature, contributing to the inhibition of LAP cleavage from TGF-*β*1 and reduction of apoptosis, could contribute to MSCs' antifibrotic effects.

### 4.4. Matrix Remodeling

Excess production of ECM and the failure to degrade it are the hallmark of fibrosis. Thus, the ultimate goal in case of fibrotic diseases is to restore a nonpathological healing process, by inhibiting ECM production and enabling the degradation of its various components. Indeed, the imbalance of MMPs, responsible for the degradation of ECM, and TIMPs, their inhibitor, results in improper ECM remodeling, hence preventing restoration to a nonpathological matrix.

In different fibrosis models, a decreased expression and concentration of collagen, the main component of the ECM, were found after MSC transplantation [41, 43–46, 48–53, 56, 58, 59, 64–68, 71, 73, 76]. This effect is also obtained after transplanting microvesicles or exosomes secreted into an MSC culture, suggesting a paracrine control of MSCs on ECM degradation [47, 69].

Changes in the expression and concentration of MMPs and TIMPs have also been studied. After MSC transplantation, the increased expression of MMP-2, MMP-9, MMP-13, and MMP-14 has been observed in several fibrosis models [48, 65, 75]. Following the addition of MSC-conditioned culture medium to a culture of heart fibroblasts, an increase in the activity of MMP-2 and MMP-9 was found [44]. Conversely, several studies have shown reduced expression, concentration, or activity of MMPs. Accordingly, Alfrano et al. noted the decreased activity of MMP-2 after transplantation in the NIRC model [64]. In some fibrosis models, MMP-2, MMP-9, and MMP-13 have a lower expression and concentration following treatment with MSCs [43, 61, 68]. However, these variations suggest restoration to levels similar to untreated controls.

MSCs seem to have a repressive effect on the expression of TIMPs such as TIMP-1 [50, 65]. A reduction in the concentration of TIMP 1 to 4 was shown after MSC transplantation [67]. In an* in vitro* model, a decrease in the expression of TIMP-2 was observed, suggesting that MSCs have a paracrine effect [44]. Finally, Linard et al. demonstrated a tendency toward the resolution of fibrosis by calculating the collagen-to-MMP-to-TIMP ratio, a marker of fibrosis evolution [40, 94].

MMP and TIMP expression are impaired in fibrotic pathologies. In fact, lower TIMP expression is generally associated with fibrosis resolution. In cases of heart failure, an increased expression of MMPs has been observed in the initial and final phase [95, 96]. It has been shown that increased MMP-2 activity is associated with pathological ECM remodeling in the kidney [97]. Thus, decreased activity following MSC therapy suggests a transition to a nonpathological state. On the contrary, it has been shown that MMP-2 is implicated in alveolar regeneration, which could explain its increased activity after transplantation in a pulmonary fibrosis model [98]. Finally, as certain MMPs activate latent TGF-*β*1, a decrease in their concentration would result in a lesser activation of downstream effectors. Taken together with a decreased fibrotic area and ECM component (collagen, fibronectin, etc.) expression, these results indicate a change in ECM composition, close to that observed in nonpathological animals. Hence, MSCs seem to improve ECM quality, allowing the appearance of a microenvironment favorable to tissue regeneration.

### 4.5. Transplantation Conditions

Various transplantation conditions have been assessed in the studies reported in this work including MSC activation and the optimization of MSC delivery. First, melatonin has been shown to improve MSC survival after transplantation, as well as having proangiogenic abilities [99, 100]. In both occurrences of this treatment, melatonin-treated MSCs exerted increased beneficial effects compared to nontreated cells, as evidenced by reduced ECM deposit and inflammation [44, 63]. Qiao et al. showed potentiation in predifferentiated MSCs treated with baicalin, which possesses anti-inflammatory and antioxidant properties [58]. Cotreatment with atorvastatin increased the survival and efficacy of MSCs [41].

Multiple transplantation timings have been compared to investigate their respective effect. Alfarano et al. showed that transplantation 7 days after ischemia-reperfusion was more effective on ECM deposition, myofibroblast proliferation, and MMP activity in their model compared to transplantation after 14 days [64]. In bleomycin-induced lung fibrosis, Ortiz et al. also observed greater effectiveness from MSCs when transplanted earlier [68].

Interestingly, Ishikane et al. demonstrated that the transplantation of fetal membrane or bone-marrow-derived MSCs yielded similar results on myocardial infarction [45].

In two different studies, the value of MSC differentiation before transplantation was observed. In the rat model of CCl_4_-induced fibrosis, opposite effects were reported. Hardjo et al. showed a higher potential for nondifferentiated MSCs, compared to adipogenic and hepatogenic differentiation, on ECM accumulation and MMP expression [57]. Conversely, in the exact same model, Qiao et al. found that hepatogenic predifferentiation had no significant influence on the effect of MSCs [58].

Recently, new delivery procedures have been studied to improve MSCs engraftment in fibrotic tissues. MSCs grown in two-layered sheets and transplanted in a rat model of myocardial infarction were found in significant number 28 days after transplantation. Part of these cells showed evidences of differentiation, participating in neovascularization of the infarct [45]. Indeed, MSC homing in the damaged tissue is generally transient, which could explain the decreased long-term benefit often observed. Embedding MSCs in scaffolds or biomaterials could improve their beneficial effects [101, 102].

## 5. MSC Clinical Trials

In clinical settings, the transplantation of MSCs has been studied on numerous pathologies. A systematic review of clinical trials evaluated the safety of MSC injections. Thirty-six studies were included representing 1012 patients. The meta-analysis did not reflect any serious complications related to MSC injections. Only a transient fever was highlighted (reviewed in [103]). Around 30 clinical trials are currently registered worldwide for evaluating MSC therapy for fibrosis (http://clinicaltrials.gov). Liver and pulmonary fibrosis are most widely represented, but some occurrences of renal and vocal fold treatment exist. MSCs engraft preferentially in the lungs and liver which is the reason for a higher number of clinical trials on these organs [104]. In most of these studies, only organ functionality is evaluated but not fibrosis markers. Thus, it is not clear whether the improvement of the symptoms and quality of life is due to fibrosis reduction or the amelioration of other pathological features.

Bone-marrow-derived MSCs improve liver function in patients with liver cirrhosis as evidenced by phase I clinical trials [105–107]. The Model for End-stage Liver Disease (MELD) score is used to evaluate the mortality risk in patients with end-stage liver disease (reviewed in [108]). The mean MELD score is significantly lower after MSC injection compared to placebo controls. In patients with decompensated liver fibrosis, MSCs significantly improved quality of life as evidenced by the increase in physical and mental component scales [105] and through the SF-36 questionnaire [109]. Inducing hepatic differentiation prior to MSC injection improved liver function in treated patients [106]. Finally, fibrosis markers were measured on 30 patients during a phase I trial [107]. Laminin, hyaluronic acid, and type IV collagen were significantly decreased 48 weeks after intervention. On the other hand, HGF, an antifibrotic growth factor, was increased after 48 weeks, as compared to nontreated patients. Based on these clinical trials [107], it appears that MSCs may exert an antifibrotic effect on liver cirrhosis.

The results of a phase I study show the ability of MSCs to reduce allograft rejection after renal transplantation [110]. MSCs decreased graft rejection by exerting immunosuppression and probably by preventing interstitial fibrosis. The absence of a placebo control in this trial did not permit the comparison and identification of the specific effect of MSCs. Thus, it is necessary to gather additional clinical data.

MSC therapy has proven to be effective in patients suffering from complications following acute myocardial infarction [111, 112]. In the first trial, functional testing showed an improvement in both heart and lung functions. There was evidence that MSC treatment led to reverse remodeling, which could be correlated with fibrosis reduction [113]. Six months after treatment, global symptom scores were significantly better in the MSC group versus the placebo group [111]. In the second study, MSC treatment reduced symptoms associated with ischemic cardiomyopathy. There was also evidence of reverse remodeling concomitant with infarct size reduction, probably linked to reduced fibrosis [112].

Pelvic radiation disease (PRD) is induced in 5 to 10% of patients within 10 years after abdominopelvic radiotherapy. Fibrosis to the colon and rectum is the main characteristic of late complications of radiotherapy. Since no satisfactory treatment exists for PRD and given the results of MSC therapy on radiation-induced burns [114], the curative potential of MSCs is being evaluated in clinical trials for PRD treatment. In particular, 4 patients suffering from serious intestinal radiation-induced lesions following overdosage of radiotherapy have been treated. The systemic administration of MSCs resulted in efficient analgesic and anti-inflammatory effects as well as hemorrhage reduction [3]. These results indicate the potential of MSC to diminish the adverse effect of radiotherapy and possibly radiation-induced fibrosis.

Based on these clinical trials, MSC therapy has proven to be safe and effective in patients suffering from diseases associated with fibrosis without the adverse effect of MSC transplantation. Nevertheless, there is a need for randomized trials (phase 3) to gather statistically significant data and to demonstrate MSCs' efficacy in limiting fibrosis.

## 6. MSC Therapy versus the Current Management of Fibrosis

The future of MSC therapy for fibrotic diseases mostly relies on a comparison with current management strategies. Results from preclinical and clinical trials highlight the ability of MSCs to act on fibrosis through different mechanisms: (i) immunosuppression, (ii) inhibition of the TGF-*β*1 pathway, (iii) reduction of hypoxia and oxidative stress, and (iv) restoration of ECM degradation. Thus, the potential of MSC therapy lies in the ability to act simultaneously on various fibrogenesis parameters. There are currently several therapy protocols for fibrotic therapies under assessment in clinical trials. Most of those treatments are designed to act on a single pathway underlying fibrosis development and progression, unlike cell therapy.

Presently, therapy protocols for fibroproliferative diseases mostly consist of symptomatic treatments. For example, patients with idiopathic pulmonary fibrosis (IPF) are often prescribed oxygen therapy and vaccination against viral and bacterial infections of the airways is recommended, if any exists. Likewise, antifibrotic strategies in the liver are most effective when they are able to cure the underlying disease. Many anti-inflammatories and antioxidants have been unsuccessful candidates for fibrosis treatment [115]. Ultimately, organ transplantation is required to ensure the survival of patients with fibrosis.

The first example of clinically used pharmacological antifibrotic agent is pirfenidone, which acts on TGF-*β*1 activity and inflammation and which has antioxidative properties [116]. It has been approved for the treatment of IPF in Europe, Canada, South Korea, and Japan. Preclinical studies have shown its ability to suppress TGF-*β*1 gene expression and to significantly reduce its concentration in lavage fluid in models of pulmonary fibrosis [117]. Pirfenidone is also effective in animal models of heart [118], kidney [119], liver [120], and radiation-induced fibrosis [121]. The FDA has not yet approved pirfenidone for pulmonary fibrosis based on a lack of efficacy and survival benefit, especially in long-term clinical trials [122]. Moreover, a meta-analysis of clinical trial results shows that pirfenidone induces adverse gastrointestinal, neurological, and dermatological adverse effects [123].

Other antifibrotic drugs are currently being examined for clinical use (reviewed in [6, 124]). Those pharmacological agents are mainly anti-inflammatory drugs and inhibitors of the TGF-*β*1 signaling pathway acting on different molecular targets. Despite the fact that some of these drugs have been evidenced to exert antifibrotic effects in animal models, there is a lack of clinical data that may lead to their approval.

Although some pharmacological compounds have proven to be effective, the necessity to use multiple drugs for the treatment of fibrosis is increasingly recognized. Furthermore, MSCs specifically home to damaged tissues and are able to behave depending on the surrounding environment, delivering transiently and locally specific molecules necessary for restoring tissue homeostasis. Conversely, drugs affect every organ, regardless of its pathological state. There is a need for more clinical data on MSC therapy to ascertain its effectiveness and safety. However, while inducing minor side effects, MSCs have shown promising antifibrotic effects, regardless of the organ, and should be considered as a major candidate.

## 7. Conclusion

Altogether, the objective analysis of the literature supports the antifibrotic effect of MSCs. It is sometimes argued that MSCs could have profibrotic properties because they are likely to acquire a myofibroblastic phenotype* in vitro* [125] or that the mesenchymal origin of myofibroblasts [126] indicates profibrotic properties. Nevertheless, there is, to our knowledge, no example showing MSC transplantation to have a profibrotic effect on a developing or established disease.

**Figure 4:**
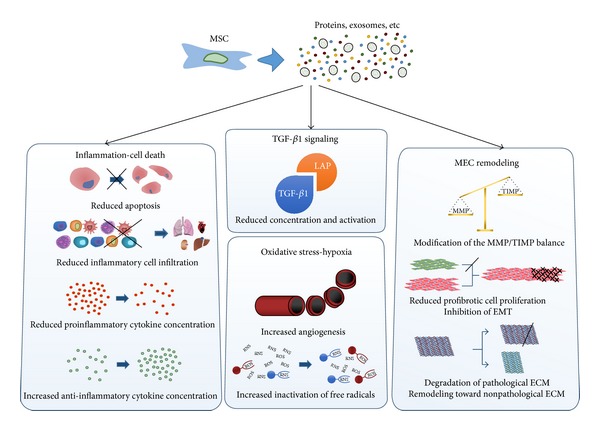
Common outcome of MSC therapy for various fibrotic diseases. Based on the studies reported in this work, several mechanisms have been underlined, mostly concerning inflammatory reaction and apoptosis, oxidative stress/hypoxia modulation, and extracellular matrix remodeling. It appears that MSC secretome activates a wide range of antifibrotic pathways (ECM: extracellular matrix, EMT: epithelial-to-mesenchymal transition, LAP: latency associated protein, MMP: matrix metalloproteinase, MSC: mesenchymal stromal cell, TGF-*β*: transforming growth factor-*β*, and TIMP: tissue inhibitor of metalloproteinase).

Since fibrosis is a very complex multicomponent process, it can be hypothesized that MSCs act through different secreted factors on multiple pathways (Figure 4). This assumption is supported by the fact that the role of transplanted MSC depends mainly on the surrounding environment.

The principal mode of action of MSCs may be exerted mainly through inhibition of the TGF-*β*1 signaling pathway, mainly by blocking the passage from its latent form to its active form. The reduction of inflammation in the tissue, the improvement of angiogenesis, and the reduced oxidative stress seem to be responsible for this effect. The decrease in the concentration of activated TGF-*β*1 would lead to reduced EMT and myofibroblast proliferation, consequently shifting the balance between synthesis and degradation of the ECM. Furthermore, results suggest that MSCs possess the ability to inhibit TGF-*β*1 mRNA as well as protein synthesis [47, 58]. Thus, they would act on two different levels, preventing injury-triggered TGF-*β*1 overexpression and modifying the surrounding microenvironment to lessen the concentration of TGF-*β*1-activating factors.

Another interesting and extensively studied feature of MSC therapy against fibrotic diseases is their immunomodulatory ability. In numerous studies reported here, MSCs seem to reduce immune cell homing in the damaged tissue [61, 70]. This could in part explain the decrease in proinflammatory cytokines mRNA expression and production. Most notably, TNF-*α* and IFN-*γ*, two major profibrotic cytokines, were underexpressed in several studies reported here [66, 67]. These observations are consistent with the implementation of an antifibrotic “virtuous circle” in which fewer immune cells migrate to damaged tissues, hence reducing proinflammatory cytokines production. By inhibiting the acute inflammatory reaction, it is conceivable that MSCs reduce the consequent chronic inflammation.

Reduced hypoxia and oxidative stress are also an important effect of MSCs in this context [52, 77]. In fact, high ROS and RNS concentrations, combined with low oxygen intake, further increase TGF-*β*1 activation. It also induces apoptosis in resident cells, resulting in increasingly elevated inflammation. The ability of MSCs to improve the neutralization of free radicals, already described in other models, is supplemented by indications of improved angiogenesis [44, 45]. The resulting improvement in tissue vasculature reduces ischemia, allowing better regeneration of the injured organ.

As expected, the inhibition of the TGF-*β*1 signaling pathway induces a substantial remodeling of the ECM toward a nonpathological state. The decreased expression and concentration of ECM components, associated with the restoration of the MMP/TIMP balance, improve the quality of the connective tissue [43, 64]. This can mostly be explained by a lower profibrotic cell population (myofibroblasts mainly). This allows for better homing of the cell types necessary for regeneration of the damaged tissue, suggesting the possibility of reversing fibrosis under the influence of MSCs.

MSCs seem to have a paracrine effect highlighted by the results obtained in studies using MSC-conditioned medium [42, 44]. Several factors have been put forward as mediating this effect. First, HGF, an antifibrotic mediator which also has antiapoptotic properties, should be mentioned. MSC therapy combined with antibodies against HGF greatly reduces the effects of the treatment and recombinant HGF administration partially reproduces the effects of MSCs [44, 62]. The treatment of fibrosis by HGF has already been assessed in earlier studies and has shown great potential [127]. Moreover, TSG-6, a recently discovered protein highlighted for its immunosuppressant effect, seems to play a major role in the antifibrotic action of MSCs [62]. The use of antibodies or gene silencing methods significantly reduces MSCs' ability to alleviate fibrosis. Indeed, TSG-6 has been demonstrated to inhibit the secretion of TNF-*α* by macrophages and to alter the TGF-*β*1/TGF-*β*3 balance toward an antifibrotic ratio [77].

The study of MSC transplantation conditions also needs extensive investigation. Data reported in this paper indicate that the pretreatment of MSCs to potentiate their effect may yield better outcomes. Equally, transplantation timing after injury is of great importance. In fact, results suggest that earlier therapies improve the efficacy of MSCs on fibrosis [49, 64]. This is to be expected, as inhibition of the acute inflammatory reaction by MSCs would prevent the onset of chronic inflammation. MSC source is also an important factor to be considered. It has been reported in this paper that fetal membrane and bone-marrow-derived MSCs were equally effective [45]. The comparison between different sources of MSCs is an important matter considering the fact that some tissues, such as adipose tissue, are easier to harvest and/or contain higher numbers of stem cells. Moreover, the value of predifferentiation is to be further investigated as contrary data have been gathered. In any case, supplementary studies need to be conducted to confirm these effects. Finally, although preclinical data suggest the strong antifibrotic effect of MSCs [41, 57, 70], most studies were carried on the early stages of fibrosis development. Since fibrosis is often diagnosed in more advanced phases, assessment of the effects of MSCs on established fibrosis is required in order to consider the routine use of MSC therapy on such pathologies.

These observations highlight the great potential of MSCs in the treatment of fibrotic diseases. Given these results, MSCs seem to act in the same way, regardless of the organ, and no occurrence of profibrotic effects has been reported. However, the mechanisms by which MSCs act on fibrosis have not yet been clearly elucidated and additional studies are needed. Besides, concerns about effects promoting certain pathologies, such as cancer, are still preventing their routine clinical use. Thus, emphasizing many pathways triggered by MSC homing is of great importance. Furthermore, the regulation of phenotypic changes in MSCs needs to be thoroughly evaluated. As described previously, exposing MSCs to profibrotic stimuli may trigger various changes in their secretome, probably leading to variable responses. Understanding the relative implication of the factors influencing MSC phenotype would provide valuable insight into potentiation and possible adverse effects. In addition, it has been shown that microvesicles or exosomes secreted by MSCs partially reproduce their effect [47, 69]. Describing their composition and elucidating the triggers influencing their content are essential. The importance of MSC homing to damaged tissues also needs to be addressed, mostly in terms of cell-to-cell contacts and microenvironment influence. Although few reports show the importance of engraftment and the differentiation of MSCs [45], these processes are likely to play a role in the beneficial effects of cell therapy. Also, optimal treatment protocols remain to be established. First, the timing of MSC transplantation surely influences the success of the therapy. The immunomodulatory effect of MSCs should in fact be most effective when transplantation is undergone during the acute inflammatory reaction to prevent the installation of chronic inflammation. Based on the results reported in this review, it is unclear whether MSCs could reverse fibrosis in its more advanced stage and fully restore tissue homeostasis. Nonetheless, MSC therapy for the treatment of fibrosis in any organ should be strongly considered and studied as it shows promising potential.

## Figures and Tables

**Table 1:** MSC treatment on preclinical heart fibrosis models.

References	Organism	Model	Treatment	MSC source	Timing	Quantity	Route	Outcome
[41]	Pig	Ligation	Ligation of the left coronary artery for 90 minutes before reperfusion	BM	30 minutes after reperfusion	3.10^7^ cells + atorvastatin	Infarction and peri-infarction zone	(i) Reduced fibrotic area (ii) Reduced inflammation score (iii) Decreased apoptosis (iv) Increased cNOS activity (v) Atorvastatin increases MSC survival

[42]	Rat	DOX	2.5 mg/kg 6 times in 2 weeks	BM	1 week after the first DOX injection	−5.10^6^ cells or −1 mL of MSC-conditioned medium	Tail vein	(i) Reduced fibrotic area (similar effect with MSC-conditioned medium) (ii) Increased Bcl-2/Bax ratio (similar effect with MSC-conditioned medium) (iii) High concentration of HGF and IGF-1 in MSC-conditioned medium

[43]	Rat	ISO	170 mg/kg every day for 4 days	BM	4 weeks after the final ISO injection	3.10^6^ cells	Intramyocardial	(i) Reduced fibrotic area. (ii) Decreased expression of type I collagen (iii) Decreased expression of pro-MMP-2, active MMP-2, and MMP-9 (iv) Decreased concentration of MMP-2 and MMP-9 (v) Increased concentration of HGF (vi) Increased expression of HGF (sham level)

[44]	Rat	Ligation	Ligation of the interventricular artery	BM (wild type or melatonin treated)	2 weeks after ligation	3 injections of 2.10^6^ cells or 3 injections of 50 *μ*L of MSC-conditioned medium	Intramyocardial	(i) Reduced fibrotic area (improved effect with melatonin) (ii) Stimulation of angiogenesis (iii) Increased concentration of HGF (improved effect with melatonin)

[45]	Rat	Ligation	Ligation of the left coronary artery	FM or BM	4 weeks after ligation	Two-layered MSC sheets	Anterior heart wall	(i) Reduced fibrotic area (ii) Increased proportion of M2 macrophages (iii) Increased concentration of VEGF (iv) Increased capillary density in peri-infarct area (v) Some MSCs engrafted 28 days after transplantation (vi) Some engrafted MSCs express *α*-SMA and/or lectin-I (vii) No significant difference between FM and BM-MSCs

Influence of fibrosis induction methods, MSC source, timing of injection, quantity of MSCs transplanted, and transplantation route. Outcomes are expressed compared to control groups (i.e., groups treated but not transplanted with MSCs) unless stated otherwise (*α*-SMA: *α*-smooth muscle actin; BAX: Bcl-2-associated X protein; Bcl-2: B-cell lymphoma 2; BM: bone marrow; DOX: doxorubicin; FM: fetal membrane; HGF: hepatocyte growth factor; IGF: insulin-like growth factor; ISO: isoproterenol; MMP: matrix metalloproteinase; MSC: mesenchymal stromal cell; NOS: nitric oxide synthase; VEGF: vascular endothelial growth factor).

**Table 2:** MSC treatment on preclinical liver fibrosis models.

References	Organism	Model	Treatment	MSC source	Timing	Quantity	Route	Outcome
[46]	Mouse	CCl_4_	1 mL/kg twice a week for 8 weeks + 100 *μ*g/kg IL-6 24 and 48 hours after completion of CCl_4_ treatment	BM	52 hours after CCl_4_ treatment completion	5 injections of 10^6^ cells	Local (liver lobes)	(i) Reduced fibrotic area (ii) Decreased apoptosis (improved by IL-6 treatment) (iii) Decreased expression of markers of apoptosis (Bax, capsase-3, and NF-*κ*B)

[47]	Mouse	CCl_4_	0.6 mL/kg twice a week in 1 week	UC	6 weeks after CCl^4^ treatment	250 *μ*g of MSC-secreted exosomes		(i) Reduced fibrotic area (ii) Decreased expression of collagens I and III (iii) Decreased expression of TGF-*β*1 (iv) Decreased concentration of TGF-*β*1 in serum (v) Decreased phosphorylation of Smad2 (vi) Decreased EMT as evidenced by the decrease in N-cadherin and vimentin positive cells

[48]	Mouse	CCl_4_	1 mL/kg twice a week for 8 weeks	BM	4 weeks after the beginning of the CCl_4_ treatment	10^6^ cells	Tail vein	(i) Reduced fibrotic area (ii) Decreased expression of *α*-SMA (iii) Decreased expression of collagen type I (iv) Increased expression of MMP-13

[49]	Mouse	CCl_4_	1 mL/kg twice a week for 2 or 5 weeks	BM	Immediately following or 1 week after CCl_4_ treatment	10^6^ cells	Tail vein	(i) Reduced fibrotic area (ii) Decreased expression of TGF-*β*1 and *α*-SMA

[50]	Mouse	CCl_4_	4 weeks CCl4 treatment followed by 4-week SNP treatment	BM	Following CCl_4_ treatment	10^6^ cells	Local	(i) Reduced fibrotic area (ii) Decreased expression of NF-kB (iii) Decreased expression of *α*-SMA (iv) Decreased expression of collagen 1*α*1 and TIMP-1 (v) Effect improved by SNP treatment

[51]	Mouse	CCl_4_	20 mL/kg twice over a 48-hour period	BM		10^6^ cells	Tail vein	(i) Reduced fibrotic area (ii) Decreased expression of *α*-SMA and collagen 1*α*1 (iii) Injection of FGF2 partially reproduces the effects of MSCs

[52]	Mouse	CCl_4_	1 mL/kg twice a week for 8 weeks	AM	After 4 weeks of CCl_4_ treatment	10^5^ cells	Spleen	(i) Reduced fibrotic area (ii) Increased hepatocyte proliferation (iii) Increased expression of Bcl-2 (iv) Decreased hepatocyte apoptosis (v) Reduced number of *α*-SMA-positive cells (vi) Increased SOD activity (vii) Increased expression of HGF and Bcl-2

[53]	Rat	CCl_4_	5 mL/kg (first injection) followed by 3 mL/kg twice a week for 12 weeks	AT	2 days after CCl_4_ treatment	2.10^6^ cells	Tail vein or hepatic portal vein	(i) Reduced fibrotic area (ii) Improvement of the microvasculature (iii) Decreased expression of VEGF

[54]	Rat	CCl_4_	0.5 mg/kg twice a week for 4 weeks	BM (wild type or HGF-treated)	Following the first CCl_4_ injection	3.10^6^ cells	Tail vein	(i) Reduced fibrotic area (improved effect after MSC pretreatment with HGF) (ii) Decreased inflammation

[55]	Rat	CCl_4_	0.08 mL/kg twice a week for 6 weeks	BM	Following CCl_4_ treatment	3.10^6^ cells	IV	Decreased collagen concentration

[56]	Rat	CCl_4_	0.5 mg/kg twice a week for 4 weeks	BM	Following CCl_4_ treatment	10^6^ cells	Hepatic portal vein	Reduced fibrotic area

[57]	Rat	CCl_4_	1 mL/kg twice a week for 8 weeks	BM (wild type or adipogenic or hepatogenic differentiation)	4 weeks after the beginning of the CCl_4_ treatment	3.10^7^ cells	Spleen injection	(i) Reduced fibrotic area (best outcome with undifferentiated MSCs) (ii) Highest expression of MMP-2 and MMP-9 after undifferentiated MSC transplantation

[58]	Rat	CCl_4_	1 mL/kg twice a week for 8 weeks	BM (wild type or hepatogenic differentiation)	4 weeks after the beginning of the CCl_4_ treatment	5.10^6^ cells	Tail vein	(i) Decreased fibrotic area (best effect with predifferentiated MSC + baicalin) (ii) Decreased concentration of TNF-*α* (best effect with predifferentiated MSC + baicalin) (iii) Decreased concentration of TGF-*β*1 (best effect with predifferentiated MSC + baicalin) (iv) Decreased collagen concentration (best effect with predifferentiated MSC + baicalin)

[59]	Rat	CCl_4_	0.5 mL/kg (first administration) followed by 1 mL/kg twice a week for 8 weeks (gavage)	UC	4 weeks after the beginning of the CCl_4_ treatment	5.10^5^ cells	Local	(i) Reduced collagen deposition (ii) Decreased concentration of TGF-*β*1 (iii) Decreased concentration of *α*-SMA (iv) Increased expression of HGF

Influence of fibrosis induction methods, MSC source, timing of injection, quantity of MSCs transplanted, and transplantation route. Outcomes are expressed compared to control groups (i.e., groups treated but not transplanted with MSCs) unless stated otherwise (*α*-SMA: *α*-smooth muscle actin; AM: amniotic membrane; AT: adipose tissue; BAX: Bcl-2-associated X protein; Bcl-2: B-cell lymphoma 2; BM: bone marrow; CCl_4_: carbon tetrachloride; EMT: epithelial-to-mesenchymal transition; FGF: fibroblast growth factor; HGF: hepatocyte growth factor; IV: intravenous; MMP: matrix metalloproteinase; MSC: mesenchymal stromal cell; NF-*κ*B: nuclear factor kappa-light-chain-enhancer of activated B-cells; SNP: sodium nitroprusside; SOD: superoxide dismutase; TGF-*β*: transforming growth factor-*β*; TIMP: tissue inhibitor of metalloproteinase; TNF-*α*: tumor necrosis factor-*α*; UC: umbilical cord; VEGF: vascular endothelial growth factor).

**Table 3:** MSC treatment on preclinical kidney fibrosis models.

References	Organism	Model	Treatment	MSC source	Timing	Quantity	Route	Outcome
[60]	Mouse	R-UUO	10 days UUO	BM	10 days after UUO	10^6^ cells	Renal artery	(i) Decreased expression of TNF-*α*, TGF-*β*1, and *α*-SMA (ii) Increased expression of E-cadherin

[61]	Pig	ARAS	Irritant coil placed in the main renal artery	AT	6 weeks after ARAS	10^6^ cells	Local	(i) Reduced fibrotic area (ii) Reduced number of CD163 + macrophages (iii) Increased number of regulatory macrophages (iv) Increased expression of IL-10 (v) Decreased expression of TNF-*α* (vi) Reduced concentration of MMP-2 (vii) Increased expression of VEGF, FLK-1, and HIF1-*α* (viii) Reduced MCP-1 positive area

[62]	Rat	Albumin-overload + uninephrectomy	Nephrectomy followed by 5 intraperitoneal injections of BSA (10 mg/g) per weeks during 4 weeks	BM	7 days after the first BSA injection	10^6^ cells weekly for 4 weeks	IV	(i) Reduced expression and concentration of MCP-1 and CCL-5 (ii) Reduced expression and concentration of *α*-SMA (iii) Reduced expression and concentration of collagen IV

[63]	Rat	Allograft	Bi-nephrectomization and single kidney allograft	BM (melatonin treated)	11 weeks after graft	5.10^5^ cells	Tail vein	(i) Decreased expression of IL-6, IL-7r, IL-23a, and IL-10 (ii) Decreased concentration of CTGF and *α*-SMA (iii) Decreased expression of fibronectin (iv) Decreased expression of bFGF

[64]	Rat	NIRC	Excision of the right kidney, 45-minutes ischemia in the left kidney followed by 28-day cyclosporine A treatment	BM	7 or 14 days after ischemia-reperfusion	3.10^6^ cells	Local	MSC transplantation 7 days after ischemia reperfusion: (i) Decreased fibrotic area (ii) Decreased expression of collagen types I, III, and IV (iii) Reduced number of *α*-SMA-positive cells (iv) Decreased activity of MMP-2 (sham level) MSC transplantation 14 days after ischemia reperfusion showed no significant improvement

[65]	Rat	RKM	5/6 nephrectomy	BM	2 weeks after surgical procedure	−2.10^5^ cells or −2.10^5^ cells every other week (weeks 2, 4, and 6)	IV	(i) Reduced fibrotic area (ii) Increased expression of IL-4 and IL-10 (iii) Decreased expression of IL-6 and TNF-*α* (iv) Decreased expression of TGF-*β*1, Smad3, *α*-SMA, FSP-1, and vimentin (v) Decreased expression of collagen type I, collagen type III, fibronectin, and TIMP-1/MMP-9 ratio (vi) Increased expression of HO-1 (vii) Decreased expression of MCP-1 (viii) Increased expression of HGF

[66]	Rat	UUO	1 to 4 weeks obstruction	BM	Prior to UUO	10^6^ cells	Renal artery	(i) Decreased collagen concentration (ii) Decreased expression of TNF-*α* (iii) Decreased concentration of TNF-*α* (iv) Decreased expression of *α*-SMA (v) Decreased number of FSP-1 positive cells (vi) Increased expression of E-cadherin

Influence of fibrosis induction methods, MSC source, timing of injection, quantity of MSCs transplanted, and transplantation route. Outcomes are expressed compared to control groups (i.e., groups treated but not transplanted with MSCs) unless stated otherwise (*α*-SMA: *α*-smooth muscle actin; ARAS: atherosclerotic renal artery stenosis; AT: adipose tissue; BSA: bovine serum albumin; CCL: chemokine ligand; CTGF: connective tissue growth factor; FGF: fibroblast growth factor; FLK: fetal liver kinase; FSP: fibroblast specific protein; HGF: hepatocyte growth factor; HIF: hypoxia-inducible factor; HO-1: heme oxygenase 1; IL: interleukin; IV: intravenous; MCP: monocyte chemoattractant protein; MMP: matrix metalloproteinase; MSC: mesenchymal stromal cell; NIRC: nephrectomy + ischemia-reperfusion + cyclosporine; R-UUO: reversible unilateral ureteral obstruction; TGF-*β*: transforming growth factor-*β*; TIMP: tissue inhibitor of metalloproteinase; TNF-*α*: tumor necrosis factor-*α*; UUO: unilateral ureteral obstruction; VEGF: vascular endothelial growth factor).

**Table 4:** MSC treatment on preclinical pulmonary fibrosis models.

References	Organism	Model	Treatment	MSC source	Timing	Quantity	Route	Outcome
[67]	Mouse	Bleomycin	0.15 mg bleomycin administered intranasally	UC	24 h after bleomycin inhalation	10^6^ cells	Tail vein	(i) Decreased expression of IL-10, IFN-*γ*, and TNF-*α* (ii) Decreased concentration of TGF-*β*1 (iii) Decreased concentration of pSmad2 (iv) Decreased expression of collagen 1*α*1 (v) Increased concentration of active MMP-2 (vi) Decreased expression of TIMP-1, TIMP-2, TIMP-3, and TIMP-4

[68]	Mouse	Bleomycin	4 U/kg bleomycin instilled in the tracheal lumen	BM	Immediately following or 1 week after bleomycin inhalation	5.10^5^ cells	Jugular vein	(i) Reduced collagen concentration (best effect when MSCs are transplanted immediately after bleomycin treatment) (ii) Decreased expression of MMP-2, MMP-9, and MMP-13

[69]	Mouse	Silica	200 *μ*g/kg twice (day 1 and 4 weeks later) by intratracheal injection	Human BM	12 and 14 weeks after first silica injection	2.10^5^ cells or 10 *μ*g of MSC microvesicles	Tail vein	Bronchoalveolar lavage: (i) Reduced number of neutrophils (ii) Reduced number of lymphocytes (iii) Reduced number of macrophages (not significant after microvesicle injection) Lung samples: (i) Reduced fibrotic area (not significant after microvesicle injection) (ii) Reduced concentration of collagen I (iii) Reduced concentration of *α*-SMA (not significant after microvesicle injection)

[70]	Rat	Bleomycin	3 mg/kg bleomycin instilled intranasally	BM	4 days after bleomycin inhalation	10^6^ cells	Tail vein	Bronchoalveolar lavage: (i) Reduced number of neutrophils (ii) Reduced number of lymphocytes (iii) Reduced number of macrophages (iv) Decreased expression of IL-6 and TNF-*α* Lung samples: (i) Reduced fibrotic area (ii) Decreased expression of IL-1*β* (iii) Decreased expression of TGF-*β* (iv) Decreased expression of VEGF (v) Decreased concentration of RNS

[71]	Rat	Bleomycin	5 mg/kg intratracheal perfusion	BM	12 h after bleomycin inhalation	5.10^6^ cells	Tail vein	(i) Reduced fibrotic area (ii) Decreased expression of TGF-*β*1 (iii) Decreased expression of PDGF-A (/1.4) and PDGF-B (iv) Decreased expression of IGF-1 (v) MSCs differentiated in alveolar epithelial cells

[72]	Rat	Bleomycin	1.28 U instilled intratracheally	BM transfected with HGF expression plasmid	7 days after bleomycin instillation	3.10^6^ cells	Intratracheal instillation	(i) Reduced Ashcroft score (fibrosis scoring) (ii) Reduced collagen concentration (iii) Transfected cells yield better results

Influence of fibrosis induction methods, MSC source, timing of injection, quantity of MSCs transplanted, and transplantation route. Outcomes are expressed compared to control groups (i.e. groups treated but not transplanted with MSCs) unless stated otherwise (*α*-SMA: *α*-smooth muscle actin; BM: bone marrow; HGF: hepatocyte growth factor; IGF: insulin-like growth factor; IL: interleukin; MMP: matrix metalloproteinase; MSC: mesenchymal stromal cell; PDGF: platelet-derived growth factor; RNS: reactive nitrogen species; pSmad: phosphorylated small mothers against decapentaplegic homolog; TGF-*β*: transforming growth factor-*β*; TIMP: tissue inhibitor of metalloproteinase; TNF-*α*: tumor necrosis factor-*α*; UC: umbilical cord; VEGF: vascular endothelial growth factor).

**Table 5:** MSC treatment on preclinical peritoneum fibrosis model.

References	Organism	Model	Treatment	MSC source	Timing	Quantity	Route	Outcome
[73]	Rat	CG	0.1% CG in 2 mL saline injected intraperitoneally	BM	30 minutes after CG injection	10^7^ cells	Intraperitoneal	(i) Decreased infiltration of monocytes/macrophages (ii) Reduced number of pSmad2 positive cells (iii) Decreased number of *α*-SMA and FSP-1 positive cells (iv) Decreased concentration of collagen I and collagen III

Outcomes are expressed compared to control groups (i.e. groups treated but not transplanted with MSCs) unless stated otherwise (*α*-SMA: *α*-Smooth Muscle Actin; BM: Bone Marrow; CG: Chlorhexidine Gluconate; FSP: Fibroblast Specific Protein; pSmad: phosphorylated Small Mothers Against Decapentaplegic Homolog).

**Table 6:** MSC treatment on a preclinical pancreas fibrosis model.

References	Organism	Model	Treatment	MSC source	Timing	Quantity	Route	Results
[74]	Rat	DBTC	8 mg/kg DBTC injected in the tail vein	UC	5 days after DBTC injection	2.10^6^ cells	Jugular vein	(i) Reduced inflammatory cell infiltration score (ii) Reduced monocyte/macrophage infiltration (iii) Reduced expression of MCP-1, VCAM-1, ICAM-1, IL-6, and TNF-*α* (iv) Reduced fibrosis score (v) Reduced expression of TGF-*β*1 (vi) Reduced concentration of collagen (vii) Reduced number of *α*-SMA-positive cells

Outcomes are expressed compared to control groups (i.e., groups treated but not transplanted with MSCs) unless stated otherwise (*α*-SMA: *α*-smooth muscle actin; DBTC: dibutyltin dichloride; ICAM: intercellular adhesion molecule; IL: interleukin; MCP: monocyte chemoattractant protein; TGF-*β*: transforming growth factor-*β*; TNF-*α*: tumor necrosis factor-*α*; VCAM: vascular cell adhesion molecule).

**Table 7:** MSC treatment on preclinical cutaneous fibrosis models.

References	Organism	Model	Treatment	MSC source	Timing	Quantity	Route	Outcome
[75]	Mouse	Bleomycin	Daily subcutaneous injection of 0.5, 1, 3, or 5 mg/mL bleomycin during 4 weeks	BM	3 hr after bleomycin injection	10^6^ cells	Local	(i) Reduced number of macrophages and neutrophils (ii) Decreased expression of TGF-*β*1 (iii) Reduced number of *α*-SMA-positive cells (iv) Decreased expression of collagen type I (v) Increased expression of MMP-2, MMP-9, and MMP-13

[76]	Mouse	Radiation-induced	35 grays irradiation	BM (autologous or allogenic)	6 weeks after irradiation	5.10^5^ cells	Tail vein	(i) No difference between autologous and allogeneic cells (ii) Reduced fibrotic area (iii) Reduced number of CD68 positive cells and CD80 positive cells (iv) Increased number of CD163 positive cells (v) Modification of macrophages toward a regulatory phenotype (vi) Increased expression of IL-10 (vii) Increased concentration of IL-10 (viii) Decreased expression of IL-1*β* and Serpine1 (ix) Decreased concentration of IL-1*α*, IL-1*β*, and TNF-*α* (x) Increased expression of PDGF-a

[77]	Mouse	Surgery	Four 6 mm full-thickness wounds on the back	BM	24 hr after surgery	10^6^ cells	Local (around the wound)	(i) Decreased concentration of TNF-*α* (sham level with TSG-6 silenced MSCs) (ii) Decreased secretion of TNF-*α* by macrophages (no change in the number of macrophages) (iii) TGF-*β*1 concentration: increased on day 2; decreased on day 5; sham level with TSG-6 silenced MSCs (iv) Increased concentration of TGF-*β*3 (sham level with TSG-6 silenced MSCs) (v) Decreased *α*-SMA expression (vi) Decreased concentration of collagens 1*α*1, 1*α*2, and 3*α*1

Influence of fibrosis induction methods, MSC source, timing of injection, quantity of MSCs transplanted, and transplantation route. Outcomes are expressed compared to control groups (i.e., groups treated but not transplanted with MSCs) unless stated otherwise (*α*-SMA: *α*-smooth muscle actin; BM: bone marrow; IL: interleukin; MMP: matrix metalloproteinase; MSC: mesenchymal stromal cell; PDGF: platelet-derived growth factor; TGF-*β*: transforming growth factor-*β*; TNF-*α*: tumor necrosis factor-*α*; TSG-6: TNF-stimulated gene 6).

**Table 8:** MSC treatment on a preclinical colorectal fibrosis model.

References	Organism	Model	Treatment	MSC source	Timing	Quantity	Route	Results
[40]	Pig	Radiation-induced	High X-ray dose (21 to 29 Grays)	BM	27, 34, and 41 days after irradiation	2.10^6^ cells	Ear vein	(i) Reduced fibrotic area (ii) Reduced leukocyte infiltration (iii) Reduced macrophages infiltration (iv) Increased M2 macrophages proportion (v) Reduced expression of iNOS (vi) Reduced expression of TNF-*α*, IL-6, and IL-8 (vii) Reduced expression of TLR-4 and TLR-5 (viii) Increased expression of IL-10 (ix) Reduced expression of col1a2 and col3a1 (x) Reduced expression of TGF-*β*1 and CTGF (xi) Decreased collagen-to-MMP-to-TIMP ratio (xii) Increased expression of VEGF in the rectal mucosa (xiii) Reduced expression of angiopoietin and PDGF in the rectal mucosa (xiv) Increased expression of eNOS, VEGF, VEGFR1, and PDGF in the colon

Outcomes are expressed compared to control groups (i.e., groups treated but not transplanted with MSCs) unless stated otherwise (BM: bone marrow; Col: collagen; CTGF: connective tissue growth factor; IL: interleukin; MMP: matrix metalloproteinase; NOS: nitric oxide synthase; PDGF: platelet-derived growth factor; TGF-*β*: transforming growth factor-*β*; TGF-*β*R: transforming growth factor-*β* receptor; TIMP: tissue inhibitor of metalloproteinase; TLR: toll-like receptor; TNF-*α*: tumor necrosis factor-*α*; VEGF: vascular endothelial growth factor; VEGFR: vascular endothelial growth factor receptor).

**Table 9:** Summary of various *in vitro* studies using MSC-conditioned medium or MSCs cocultured with cells of interest.

References	Culture conditions	Cell type	MSC source	Outcome
[44]	MSC-conditioned medium	Cardiac fibroblasts	BM	(i) Reduced collagens I and III deposit (ii) Decreased viability (iii) Decreased expression of *α*-SMA (iv) Increased of MMP-2 and MMP-9 activity (v) Increased expression of MT1-MMP (vi) Decreased expression of TIMP-2 MMP-2 −/− MSC-conditioned medium: (i) No change in collagen concentration Incubation with anti-HGF antibody: (i) Reduced MMP-2 and MMP-9 activity (ii) Decreased expression of MMP-2 (iii) Increased expression of TIMP-2

[64]	MSC-conditioned medium	TGF-*β*1-treated HK2	BM	(i) Decreased concentration of *α*-SMA (ii) Increased concentration of E-cadherin

[51]	Coculture: MSCs	Fibrotic hepatocytes	BM	Increased secretion of FGF2

[73]	Coculture: MSCs in Transwell	HPMCs	BM	(i) Decreased expression of TGF-*β*1 (ii) Decreased expression of fibronectin (iii) Decreased concentration of pSmad2 (iv) Decreased expression of *α*-SMA

[77]	Coculture LPS + IFN-*γ*-treated MSCs	Activated macrophages	BM	(i) Reduced concentration of TNF-*α* and IL-12 (ii) Reduced concentration of NO

[62]	MSCs in Transwell Pretreatment of one or both cell types with HSA	PTECs	BM	(i) Reduced expression of TNF-*α*, IL-6, IL-8, MCP-1, and CCL-5 (ii) Inhibition of NF-*κ*B nuclear translocation (iii) Reduced EMT (iv) Increased expression and concentration of HGF and TSG-6 by MSCs exposed to HSA (v) Recombinant HGF or TSG-6 partially reproduces MSCs'effects

Influence of culture conditions on the outcome. Outcomes are expressed compared to control groups (i.e., groups treated without the use of MSC treatment) unless stated otherwise (*α*-SMA: *α*-smooth muscle actin; BM: bone marrow; CCL: chemokine ligand; EMT: epithelial-to-mesenchymal transition; FGF: fibroblast growth factor; HGF: hepatocyte growth factor; HK2: human kidney 2; HPMC: human peritoneal mesothelial cells; HAS: human serum albumin; HGF: hepatocyte growth factor; IFN-*γ*: interferon-*γ*; IL: interleukin; LPS: lipopolysaccharide; MCP: monocyte chemoattractant protein; MMP: matrix metalloproteinase; MSC: mesenchymal stromal cell; NF-*κ*B: nuclear factor kappa-light-chain-enhancer of activated B-cells; NO: nitric oxide; proximal tubular epithelial cell; pSmad: phosphorylated small mothers against decapentaplegic homolog; TGF-*β*: transforming growth factor-*β*; TIMP: tissue inhibitor of metalloproteinase; TNF-*α*: tumor necrosis factor-*α*; TSG-6: TNF-stimulated gene 6).

## Abbreviations

5/6 NX: 5/6 nephrectomy
*α*-SMA: *α*-Smooth muscle actin
AM: Amniotic membrane
ARAS: Atherosclerotic renal artery stenosis
AT: Adipose tissue
BAX: Bcl-2-associated X protein
Bcl-2: B-cell lymphoma 2
BM: Bone marrow
BSA: Bovine albumin serum
CAN: Chronic allograft nephropathy
CCL: Chemokine ligand
CCl_4_: Carbon tetrachloride
CG: Chlorhexidine gluconate
CKD: Chronic kidney disease
Col: Collagen
CsA: Cyclosporine A
CTGF: Connective tissue growth factor
DBTC: Dibutyltin dichloride
DC: Dendritic cell
DOX: Doxorubicin
ECM: Extracellular matrix
EMT: Epithelial-to-mesenchymal transition
EndMT: Endothelial-to-mesenchymal transition
EPS: Encapsulating peritoneal sclerosis
FGF: Fibroblast growth factor
FLK: Fetal liver kinase
FM: Fetal membrane
FSP: Fibroblast specific protein
GPx: Glutathione peroxidase
Gr: Glutathione reductase
HGF: Hepatocyte growth factor
HIF: Hypoxia-inducible factor
HK2: Human kidney 2
HLA: Human leukocyte antigen
HO-1: Heme oxygenase 1
HPMC: Human peritoneal mesothelial cells
HSA: Human serum albumin
HSC: Hepatic stellate cell
ICAM: Intercellular adhesion molecule
IDO: Indoleamine 2,3-dioxygenase
IFN-*γ*: Interferon-*γ*
Ig: Immunoglobulin
IGF: Insulin-like growth factor
IL: Interleukin
IPF: Idiopathic pulmonary fibrosis
ISO: Isoproterenol
IV: Intravenous
LAP: Latency associated protein
LPS: Lipopolysaccharide
MCP: Monocyte chemoattractant protein
MI: Myocardial infarction
MMP: Matrix metalloproteinase
MSC: Mesenchymal stromal cell
NF-*κ*B: Nuclear factor kappa-light-chain-enhancer of activated B-cells
NIRC: Nephrectomy + ischemia-reperfusion + cyclosporine
NK: Natural killer
NO: Nitric oxide
NOS: Nitric oxide synthase
NQO1: NADPH quinone oxidoreductase 1
Nrf2: Nuclear factor (erythroid-derived 2)-like 2
PDGF: Platelet-derived growth factor
pSmad: Phosphorylated small mothers against decapentaplegic homolog
PGE2: Prostaglandin E2
PTEC: Proximal tubular epithelia cell
RKM: Remnant kidney model
RNS: Reactive nitrogen species
ROS: Reactive oxygen species
R-UUO: Reversible unilateral ureteral obstruction
Smad: Small mothers against decapentaplegic homolog
SNP: Sodium nitroprusside
SOD: Superoxide dismutase
Tc: Cytotoxic T-cell
TGF-*β*: Transforming growth factor-*β*
Th: Helper T-cell
TGF-*β*R: Transforming growth factor-*β* receptor
TIMP: Tissue inhibitor of metalloproteinase
TLR: Toll-like receptor
TNF-*α*: Tumor necrosis factor-*α*
Treg: Regulatory T-cell
TSG-6: TNF-stimulated gene 6
UC: Umbilical cord
UUO: Unilateral ureteral obstruction
VCAM: Vascular cell adhesion molecule
VEGF: Vascular endothelial growth factor
VEGFR: Vascular endothelial growth factor receptor.
